# Contextual factors influencing schistosomiasis treatment and identification of delivery platforms for arpraziquantel in hard-to-reach areas and populations in Homa Bay County, Kenya

**DOI:** 10.1371/journal.pgph.0004035

**Published:** 2024-12-19

**Authors:** Phyllis Munyiva Isaiah, Doris Osei Afriyie, Mary Maghanga, Donna Obare Ogeto, Mary Amuyunzu Nyamongo, Peter Steinmann

**Affiliations:** 1 Swiss Tropical and Public Health Institute, Allschwil, Switzerland; 2 University of Basel, Basel, Switzerland; 3 African Institute for Health and Development, Nairobi, Kenya; 4 Division of Vector Borne and Neglected Tropical Diseases, Ministry of Health, Nairobi, Kenya; PLOS: Public Library of Science, UNITED STATES OF AMERICA

## Abstract

A new formulation of praziquantel, arpraziquantel (arPZQ), has been developed for preschool-aged children (PSAC) to fill the treatment gap for this age group in schistosomiasis control and elimination programs. There is now a priority to ensure that the drug reaches all at-risk PSAC in endemic areas, including hard-to-reach areas and populations. This study aimed to determine schistosomiasis treatment-related contextual factors among fishermen and island populations in Homa Bay County, Kenya, and to identify a suitable platform to deliver arPZQ. We conducted a generic qualitative study using two case study interviews with parents/caregivers living with disability caring for children ≤5 years,18 focus group discussions (FGDs) with parents/caregivers of children ≤5 years (each with 8–10 participants), 14 key informant interviews (KIIs) with various government agencies, and unstructured observations. The data were analyzed using thematic analysis. The case study interviews and FGDs revealed awareness of schistosomiasis among community members but limited knowledge of transmission risk factors. Lake water and open defecation were the main predisposing factors to infection. We observed poor health-seeking behavior in the community due to inaccessibility of quality healthcare services, resulting from health system level, population level, and geographic barriers. Despite these barriers, community members reported positive experiences with previous PZQ mass drug administration (MDAs) and other innovative healthcare programs, and expressed willingness to participate in future MDAs, including with arPZQ. Based on the reported barriers, the door-to-door distribution approach by community health promoters was proposed by parents and key informants as the most feasible platform for community sensitization, mobilization, and arPZQ delivery. To achieve high arPZQ treatment coverage for all at-risk PSAC, and promote ownership and sustainability of the program, the door-to-door approach is the most promising platform to deliver treatment and public health promotion in marginalized hard-to-reach island populations of Lake Victoria, Kenya.

## Introduction

Schistosomiasis is a neglected tropical disease (NTD) with a considerable public health impact in sub-Saharan Africa [1]. The disease affects poor rural populations in the vicinity of stagnant water bodies, especially agricultural and fishing communities without access to safe water and proper sanitation facilities [2]. *Schistosoma mansoni* and *Schistosoma haematobium* are the main species infecting humans in Africa [3] where ninety percent of the 251 million affected people in need of treatment live [2]. In Kenya, both *S*. *mansoni* and *S*. *haematobium* are endemic in many parts of the country. The World Health Organization (WHO) estimates that over 9.1 million Kenyans are infected with schistosomes [4]. The disease can lead to anemia, reduced ability to learn, chronic inflammation of organs, and stunting in children [2, 5]. If left untreated, chronic schistosomiasis can progress to liver, spleen, and kidney damage, bladder cancer as well as poor sexual and reproductive health in adulthood [6]. Over the years, across endemic countries, schistosomiasis control has focused on the periodic administration of praziquantel (PZQ), an intervention termed Mass Drug Administration (MDA), to school-aged children (SAC) and sometimes adults in high endemic areas where the prevalence among SAC is ≥ 50% [4].

Despite an estimated 50 million preschool-aged children (PSAC) in Africa being infected with schistosomes [7], PSAC are largely left out of control programs [8]. In the past, this was mainly due to limited data on the safety of PZQ among children below four years [9, 10], PZQ’s bitter taste and large tablet size [11], and the categorization of PSAC as a low-risk group for schistosomiasis [12]. In recent years, there has been more research on schistosomiasis and the morbidity it causes among PSAC, revealing an important disease burden and treatment gap previously overlooked [13, 14]. A systematic review quantifying schistosomiasis burden in PSAC living in sub-Saharan Africa for the period 2000–2020, by Kalinda *et al* [15], reported an average prevalence of 22% for *S*. *mansoni* by Kato-Katz and 15% for *S*.*haematobium* by urine filtration in 16 countries. In Ghana, children as young as four months were found to be infected [16]. In Uganda, the national *S*. *mansoni* prevalence was estimated at 25.6% (95% CI: 22.3, 29.0), N = 9,183, with children 2–4 years most at risk for schistosomiasis. Among this sub-group, 36.1% (95% CI: 30.1, 42.2) were infected [17]. Another review on the epidemiology of pediatric schistosomiasis with a focus on hard-to-reach areas and populations [18] documented a high prevalence of *S*.*mansoni* (12.9–90.5%) by Kato-Katz and point-of-care circulating cathodic antigen (POC-CCA) in hard-to-reach fishing populations. Additionally, the review documented morbidity among PSAC in hard-to-reach areas and populations, specifically the association between schistosomiasis and anemia, hematuria, and anthropometric derangements.

Efforts to address the aforementioned treatment gaps among PSAC have resulted in the development of a new pediatric formulation of praziquantel, namely arpraziquantel (arPZQ), by the Pediatric Praziquantel Consortium [19]. The new formulation is a small tablet (150 mg), has an acceptable taste, and is suitable for storage and use in tropical climates. The efficacy, safety and palatability of arPZQ was determined in an open-label, partly randomized, phase 3 trial in Côte d’Ivoire and Kenya [20]. The pediatric praziquantel consortium is currently facilitating the introduction and adoption of arPZQ in endemic countries.

It is critical to expand the current treatment strategy to include all populations in need of treatment, with the aim of achieving treatment equity, which is currently not attained for NTDs [17, 21]. Hard-to-reach areas and populations are disproportionately vulnerable to infectious diseases, including schistosomiasis, and are often insufficiently covered by health interventions. This limited service coverage is due to barriers including poor health infrastructure that does not meet their needs, physical and geographic shortcomings, and socio-economic and cultural barriers [22]. Hard-to-reach populations include ethnic minorities, nomadic and migrant populations, fisherfolk, and other minority or marginalized populations [18]. Little is known about the contextual factors that may influence the new formulations’ acceptability and the most feasible delivery approach for arPZQ in hard-to-reach populations.

This study sought to understand schistosomiasis treatment-related contextual factors in specific sub-populations, and aimed to identify a suitable platform to deliver arPZQ for PSAC in hard-to-reach island populations of Lake Victoria in Homa Bay County, Kenya.

## Methods

### Ethical considerations

This study is part of a larger research protocol that has been approved by the Ethikkommission Nordwest- und Zentralschweiz (EKNZ), (ID A0_2023–00050), Switzerland. In Kenya the study data was collected in the context of a larger study on preparing for the adoption and delivery of the arPZQ to PSAC by the Pediatric Praziquantel Consortium that has been approved by Kenya Medical Research Institute (KEMRI’s) Scientific and Ethics Review Unit (SERU), (SERU No.4375).

Community sensitization was conducted in June 2023 before data collection to explain the aim, procedures and expected outcomes of the study to key informants and parents/guardians of PSAC. The latter then granted written informed consent prior to inclusion in the study.

In this manuscript, we have omitted the names of study participants to protect their confidentiality and anonymity.

### Inclusivity in global research

Additional information regarding the ethical, cultural, and scientific considerations specific to inclusivity in global research is included in the supporting information (S1 Checklist).

### Study setting and context

Kenya is a country located in the eastern part of the African continent. It is bordered in the North by Ethiopia, in the East by Somalia, in the West by Uganda, in the South by Tanzania and in the southeast by the Indian Ocean.

**Fig 1:**
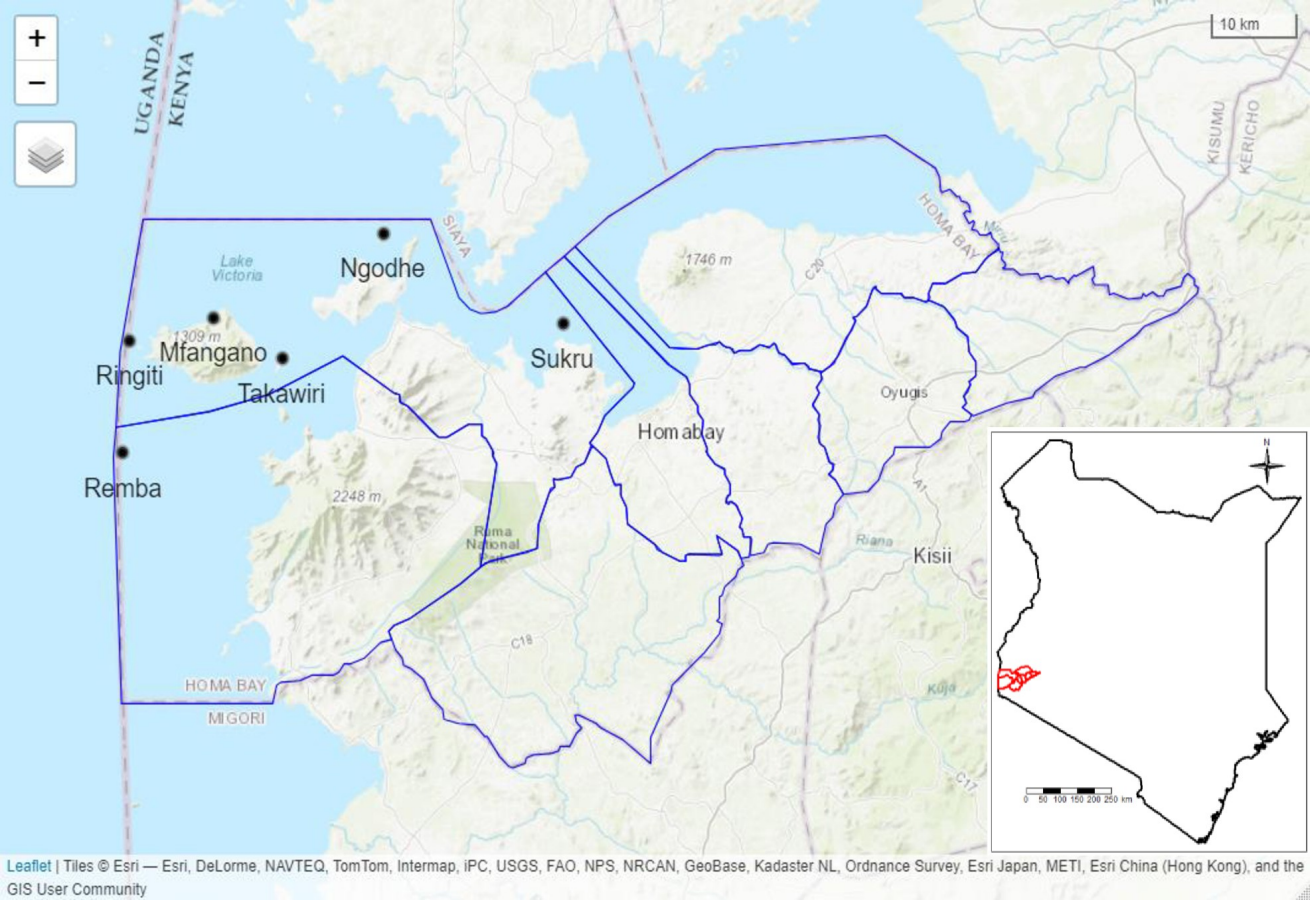
Map of the study area. The highlighted borders outline Homa Bay County. Basemap shapefile source: https://www.esri.com/en-us/arcgis/products/arcgis-location-platform/services/basemaps.

This study was conducted in Homa Bay County (Fig 1), which is located on Lake Victoria at 0°25’S (latitude) and 34°12’E (longitude) [23]. We focused on 6 islands in two sub-counties of Homa Bay County: Suba North and Suba Central (commonly known as Mbita sub-County). All six islands in the area are considered hard-to-reach [24] and were selected for this study: Sukru and Ngodhe islands are located in Suba North, while Takawiri, Remba, Mfangano, and Ringiti islands belong to Suba Central.

The study area has 84 villages with about 6,000 households, a total population of 32,000 and an average household size of 5.3 individuals [25]. Most of the Mbita population and islanders live along Lake Victoria in beach communities, and the main economic activity is fishing. In addition, households use the lake for personal hygiene, household item cleaning, irrigated farming, and recreational purposes. Houses in the study area are mainly traditional huts made from mud and small rectangular houses made of iron sheets. Open defecation is prevalent in the area. Despite many non-governmental development initiatives in the area, poverty remains a significant challenge, with over three-quarters of the population living on less than 1 USD/day (the World Bank’s definition of extreme poverty) [26]. Besides Sukru Island, all the others have health facilities and early childhood development (ECD) centres within a radius of five kilometres. However, the health facilities and ECD centres are inaccessible to most of the community due to the hilly terrain, limited transportation options, financial constraints, and service interruptions. Each island is covered by a Community Health promoter (CHP) who provides promotive, preventive, basic curative, and rehabilitative services as guided by the Kenya Community Health Strategy 2020–2025 [27].

The islands are highly endemic for schistosomiasis with *S*. *mansoni* being the predominant species (prevalence 60.5% in SAC) [24, 28, 29]. Schistosomiasis control in the area has focused on mass administration of PZQ to SAC through the Kenya National School-based Deworming Programme since 2012 [30]. In 2019, the Division of Vector Borne and Neglected Tropical Diseases, Ministry of Health, Kenya initiated a schistosomiasis community-based deworming program in the area targeting SAC and adults.

In this study, a modified definition of hard-to-reach areas and populations as defined in WHO’s ‘*Microplanning for immunization service delivery using the Reaching Every District (RED) strategy* [31] was used, as follows: (i) rural hard–to–reach: rural populations with no or irregular contact with health services. This comprises nomadic populations and people living too far from health services due to physical distance or (and) inadequate logistic and human capacity; and (ii) socio-economic hard-to reach: marginalized groups who do not use health services due to political, economic, and social reasons.

### Study design and data collection

The data for this study was collected from 20^th^ July to 4^th^ August 2023, after a community sensitization exercise in June 2023, in the context of a larger study on preparing for the delivery of the arPZQ to PSAC by the Pediatric Praziquantel Consortium [32]. Our study was a cross-sectional generic qualitative study [33–36] that employed several qualitative tools including; case study interviews, Focus Group Discussions (FGDs), Key Informant Interviews (KIIs), and unstructured observations in the study communities. The aim was to understand the context, assess parents’/caregivers’ knowledge, attitudes and perceptions towards schistosomiasis and interventions, and propose a suitable approach to deliver pediatric praziquantel (arPZQ) to hard-to-reach areas and populations.

Semi-structured FGD, case study and KII guides were designed to explore patterns and perceptions of schistosomiasis, treatment programs in the community, the preferred method of treatment delivery, gatekeepers in the community, and available healthcare infrastructure. The guiding questions used in this study were adapted from an extensive study conducted by the Pediatric Praziquantel Consortium [32].

Focus group discussions with parents/caregivers of PSAC (under 5 years) were conducted in 14 randomly selected villages. To ensure adequate representation of the study area, 14 villages were identified out of the total 84 villages in the study area with selection probability proportional to the population size across all six islands of Suba North and Suba Central sub-counties (Table 1).

**Table 1:** Study sites of interviewed participants.

Study Islands	Study villages
Mfangano	Kandega B Kiwari B Gonda B Kiduwa
	Kaswaga Nyasumbi
	Uzuwi
	Wamai East
Ringiti	Wuoth Ogik
	Koywai
Remba	Kodianga B
	Sango CBD A
Takawiri	Singla
Sukru	Sukru
Ngodhe	Ngodhe South

18 FGDs with male and female parents/caregivers of children aged ≤5 years (each with 8–10 participants) were conducted. The age of men and women who participated in the FGDs ranged between 24–53 years and 18–48 years, respectively. Most participants had at least a primary school education, with men having on average more years of formal schooling than women. Over half of the men worked as fishermen and trading was the most popular occupation for women. Participants were mainly from western Kenya and identified as Christians, except in Remba and Ringiti islands, which have a mixed community with residents from several East African countries, mostly Muslims. The demographic characteristics of the FGDs’ participants is summarized in the Table 2 below. Two case study interviews were conducted with parents living with disability (one male and one female, aged 42 and 27 years respectively) to identify how disability may influence access to health information and services. The parents/caregivers included in the FGDs and case study interviews were selected based on their availability (convenience sampling).

**Table 2:** Demographic characteristics of FGD study participants.

Demographics of FGDs participants		
Variables	Men	Women
**Gender**	48% (80/167)	54% (87/167)
Age range (years)	24–53	18–48
**Education**		
None	-	22% (19/87)
Primary	30% (24/80)	38% (33/87)
Secondary	50% (40/80)	38% (33/87)
Tertiary	20% (16/80)	2% (2/87)
**Religion**		
Christianity	86% (69/80)	83% (72/87)
Islam	14% (11/80)	17% (15/87)
**Occupation**		
Fishermen	58% (46/80)	13% (11/87)
Traders	11% (9/80)	34% (30/87)
Farmers	26% (21/80)	20% (17/87)
Formal employment (teachers, clerks)	1% (1/80)	1% (1/87)
Unemployed	4% (3/80)	32% (28/87)

All FGDs and case study interviews were conducted in Dholuo, the local language of the study area, by four trained research assistants—two males (both public health professionals) and two females (a public health officer and a community health nurse)—from the study area. The interviewers were fluent in Dholuo, Swahili, and English. FGDs and case study interviews with men were conducted by the male interviewers, and FGDs and case study interviews with women were conducted by the female interviewers. Each interview took between 30 and 60 minutes.

Key informant interviewees were purposively selected among health authorities at the County and sub-County levels and local administrative representatives. The selected participants comprised the health director, NTD coordinators, health promotion officer, WASH coordinator, school health coordinator, community services coordinator, immunization services coordinator, nutrition coordinator, malaria coordinator, clan elders, and Beach Management Unit (BMU) representatives. The BMU is an organization of fisherfolk at the lake shore (boat crew, boat owners, managers, charterers, fish processors, fishmongers, local gear makers or repairers, and fish equipment dealers) within a fishing community, mandated by the government of Kenya to support fisheries management and enhance community livelihood in an effective, efficient and sustainable manner [37]. A total of 14 KIIs were conducted in English by three of the authors (PMI, DOO, and MM). Each interview took between 30 and 60 minutes.

In addition, all interviewers took field notes throughout the study period to document unstructured observations of the daily life and behaviours of community members and the study area settings. At the end of each day, each interviewer outlined a daily diary of what they observed, experienced during the day and also described their experiences during the interviews. This was to ensure transparency in both the participants’ perspectives and the researchers’ influences. Additionally, these diary entries were reviewed and discussed with research team during team meetings. The insights gathered from these descriptions were factored into the data analysis process.

### Data analysis

The interviews were audio-recorded and transcribed. FGDs and case study interviews were then translated from Dholuo to English by the interviewers. All transcripts and diary notes were read several times to understand the collected information, after which they were then imported to Nvivo 14 software (Lumivero) for analysis. The data was coded using qualitative thematic analysis utilizing the dualistic technique of inductive and deductive thematic analysis which involves the development and description of an analytical codebook, as outlined by Fereday and Muir-Cochrane [38]. The deductive analysis approach was used to develop a preliminary codebook based on our research questions, existing literature, and preliminary scan of the raw interview files and notes [39], while the inductive approach allowed for enrichment of the codebook by allowing unexpected themes to be generated during the coding process [40].

Two authors (PMI and DOA) coded the data separately to generate an initial list of codes before validation by intercoder agreement. The codes were then grouped into categories which were used to generate themes. The Table 3 below shows categories and themes generated from coding our study data.

**Table 3:** Themes and categories.

Theme	Category
Healthcare context of Suba North and Suba Central sub-counties in relation to schistosomiasis.	Knowledge of schistosomiasis in hard-to-reach islands and populations of the study area.
	Predisposing factors to schistosomiasis in hard-to-reach communities.
	Practices around health care seeking in hard-to-reach communities.
	Barriers to accessing health care services in hard-to-reach communities.
	Previous experiences with health interventions.
Feasible arPZQ delivery and social mobilization platforms.	Preferred arPZQ delivery platforms.
	Preferred social mobilisation platforms.
Community recommendations for a successful delivery of arPZQ.	Health promotion on risks and control of schistosomiasis
	Timing of arPZQ distribution
	Expanding arPZQ distribution days and involving more CHPs.
	Integration of arPZQ within existing platforms/programs.

## Results

### Healthcare context in relation to schistosomiasis

#### Knowledge of schistosomiasis in the hard-to-reach islands/populations of Suba North and Suba Central sub-counties

The FGDs and case study interviews revealed the spectrum of understanding about schistosomiasis among community members. Nearly all community members interviewed demonstrated at least some knowledge of the common symptoms of schistosomiasis, such as fever, diarrhea, abdominal pain, vomiting, fatigue, loss of appetite, weight loss, stunted growth, and blood in stool or urine. In Dholuo language, schistosomiasis is referred to as *‘Alaremo/ ‘Layo remo’*, which loosely translates to ‘blood in urine,’ an indication of chronic urogenital schistosomiasis in the area.

*“It is called “Alaremo” blood in urine*, *but sometimes the blood can also be seen in stool*.*” (Female FGD*, *Ngodhe)*

Community feedback during the FGDs revealed a widespread awareness that schistosomiasis can affect individuals of all ages, including preschoolers, school-going children, and adults. Additionally, knowledge about prevention measures against schistosomiasis was evident. Most of the participants recognized the importance of deworming, personal and environmental hygiene practices, and the use of personal protective equipment (PPE) when undertaking water-related activities. Most of the participants were aware of PZQ, the drug used in MDAs for the treatment of schistosomiasis and prevention of morbidity, and the drug’s potential transient side effects like weakness, nausea, vomiting, sweating, stomach upset, and diarrhea.

*“This disease infects everybody regardless of age*.*” (Female FGD, Kiwari-Mfangano)*.

*“Deworming can be a good option*.*” (Male FGD*, *Kodianga B- Remba)*

*“Another preventive measure is that we should always use the toilets and avoid urinating in water or defecating in the bush*.” *(Female case study*, *Ngodhe)*

*“… use of protective gears like gumboots and the rubber gloves to avoid contact with dirty water*.*” (Male FGD*, *Gonda- Mfangano)*

*“I really had experienced the effects [PZQ side effects]… diarrhea*, *vomiting*, *and abdominal pain …” (Female FGD*, *Ngodhe)*

The majority of the participants had limited knowledge of the specific transmission mechanisms of schistosomiasis. Nearly all community members associated schistosomiasis with drinking lake water that is not boiled. There was also widespread belief that schistosomiasis transmission occurs through skin pores or only when urinating in the lake.

*“…we get it from water; we are consuming unsafe*, *water that is not boiled*.*” (Female FGD, Ringiti)*

*“…it can be contracted when someone urinates while inside the water*. *As the person passes the urine*, *the worm may get a chance of penetrating through the urinary organ into the body*.*” (Male FGD*, *Soklo- Mfangano)*

Although hematuria (blood in urine) is a symptom for urogenital schistosomiasis, community members had limited knowledge on this. They were more familiar with other possible causes of hematuria, such as sexually transmitted infections (STIs) like syphilis and gonorrhea. Some respondents believed that hematuria resulted from drug interactions between PZQ and antiretroviral drugs. Few participants mistook schistosomiasis for amoebiasis or “*Nyaldiemo*” (cholera).

*“…Sometimes we assume the symptoms*, *and take it as Sexually Transmitted Infection (STI) and get ashamed to go for medical treatment*.*” (Female FGD, Sukru)*

*“Just like I told you*, *the bigger population in Ringiti are under care [Using ARVs]*. *How do the bilharzia drugs react to those under care [Using ARVs] as opposed to the healthy people*?*” (Male FGD*, *Ringiti)*

*“What I know that differentiating bilharzia from amoeba is difficult because all present with stomach pains*. *For me*, *I know they are different*, *but the people around always mistake bilharzia for amoeba*.*” (Male FGD*, *Kodianga B- Remba)*

#### Predisposing factors to schistosomiasis in hard-to-reach communities

A key factor observed during visits to the communities and the interviews was that children often played in the lake while caregivers worked, increasing their risk of schistosomiasis due to contact with contaminated water. The lake’s accessibility and attraction as a recreational sport for children make it difficult to reduce this risk.

*“… here even the children of 2 years*, *you will find them in the lake [playing] because most of the activities are around the lake*, *so the chances of children being infected [with schistosomiasis] are high*.*” (KII*, *sub-county public health officer)*

The absence of proper sanitation facilities, particularly the lack or inaccessibility of toilets for community members emerged as a significant predisposing factor. All islands had identifiable open fields for open defecation, commonly referred to as “Mabanga,” not far from the lake.

*“Mabanga is a field just left for open defecation*.*” (Male FGD*, *Kodianga B- Remba)*

#### Practices around health care seeking in hard-to-reach communities

Most participants reported a tendency to observe a child’s health condition before deciding on a course of action when the child is sick. Participants mentioned various healthcare options, from formal medical facilities to traditional health practices. CHPs were the top choice, due to their proximity to the community members. The discussions indicated that they are trusted and respected within their communities.

*“I go to our CHV and ask her to come and examine my child*.*” (Female FGD*, *Takawiri)*

*“…*.*CHPs are well known to these people [community members] and from my experience with CHPs*, *nobody resists their approach in the community*, *they [community members] prefer them to facilities*.*”(KII*, *county NTD coordinator)*

Parents mentioned they typically visited public health centers for childhood severe illnesses or when treatment was financially viable. They often relied on over-the-counter medicines or used medications prescribed for other conditions or other family members and neighbors. Spiritual practices like prayers were also part of the health seeking approach. Additionally, some community members turned to witch doctors for traditional healing, indicating the diversity of treatment avenues. The health workers also confirmed the community’s limited appropriate health-seeking behavior.

*“I can say one of the challenges is that health-seeking behavior is very poor*. *Most of the households will only come to the hospital when they are sick*, *and this is causing a challenge with our children like for growth monitoring*. *We are supposed to have the children assessed every month until they are age 5 years*, *but this can’t happen because once they get to their last vaccinations they stay away … and they will only come if the child is sick*. *…‥”(KII*, *county nutritionist)*

Mothers were viewed as the primary decision-makers for their children’s health due to their closer proximity and better understanding of their needs. Fathers also play a role, but less frequently. Some families made healthcare decisions together, with both parents contributing.

*“Mostly*, *it is mothers because they spend more time with the children*. *For us men*, *we will only come in when finances are involved*.*” (Male FGD*, *Takawiri)*

#### Barriers to accessing healthcare services in hard-to-reach communities

Our study identified three barriers to accessing healthcare services in the six islands of Mbita. These were health system level, population level, and physical geographic barriers.

On the health system level barriers, participants raised concerns on three main issues.

- *Quality and availability of services in health facilities*. Nearly all participants reported a lack of medicines, laboratory facilities, and other supplies in public health facilities. They also expressed concerns about the unavailability of health services at all times, coupled with understaffed and unmotivated workers in the public health facilities.
  *“…Takawiri health facility is understaffed*, *and there is not enough medicine*. *We only have one nurse (in charge)*, *and if he is engaged in other duties*, * there is no treatment at the facility*, * forcing people to travel to either Mbita or Sena sub-county hospitals to seek medical attention (which is expensive due to the transportation involved)*.*” (Male FGD*, * Takawiri)*
- *Inadequate community mobilization for health interventions*. Most participants reported a lack of prior awareness before health interventions, including schistosomiasis control activities, were conducted in their areas.
  *“… We normally face a lack of mobilization before distribution of drugs.” (Female case study*, *Sukru)*
  *“For me*, *I feel the main challenge we have here is always a lack of adequate creation of awareness in various campaign programmes*. *During the recent [PZQ] MDA*, *I just took the drug but I was somehow hesitant because I just met one of the distributors who measured my height and just gave me the drugs without proper and elaborate explanation*.*” (Male FGD*, *Wuoth Ogik)*
- *Low political will in ensuring equitable health care in the area*. A major impediment identified for both the county and sub-county health management teams was a lack of resources and funding for the health interventions in the area, to reach all those targeted. Most of our KIIs also revealed that the available resources in the area mainly focus on other priority diseases compared to NTDs.
  *“There’s a lot of support on malaria*, *HIV*, *COVID*, *but when it comes to neglected [diseases] like bilharzia… it’s silent*.*” (KII*, *sub-county NTD coordinator)*

On the population level barriers, socio-economic status of the communities in the study area played a key role in their ability to access healthcare services. Low income, myths, cultural and religious hindrances were the most prominent barriers to healthcare access. Some participants felt that they were discriminated and left out of government health services.

*“…in this community*, *there are people who cannot afford daily meals and medical bills……we are a marginalized group*, *and in most cases*, *we miss services like health cover and insurance*.*” (Male case study*, *Ngodhe)*

Physical and geographic barriers in the area were due to lack of proper road networks, hilly and rocky terrains, and inadequate water transport. Additionally, the available public health centers were approximately over 5km from the villages, making it difficult for the island groups to freely access healthcare services.

*“The distance and terrain from homes to the health facilities is far*, *making accessibility to the health centers a challenge*.*” (Male FGD*, *Uzuwi)*

#### Previous experiences with health interventions

Community members who had participated in previous deworming programs reported positive feedback, highlighting the benefits of such programs. Additionally, most KII participants reported high uptake and positive experiences with new intervention programs, such as the malaria vaccine. This positive feedback reflected the communities’ willingness to embrace and benefit from innovative healthcare initiatives.

*“Based on the benefit I have seen from this drug*, *my younger daughter*, *who is four years old*, *had worms and took this drug for the first time in the past*, *and the second time was recently*, *but I can see a great change in her*. *This means the drug helped her*.*” (Male FGD*, *Gonda)*

*“… the malaria vaccine was introduced not long ago*, *and already is doing well*. *Most of the parents are embracing it because they are seeing the cases of malaria reduced in most of our communities*.*” (KII*, *county nutritionist)*

### Feasible arPZQ delivery and social mobilization platforms

#### Preferred arPZQ delivery platforms

Nearly all participants preferred the door-to-door delivery approach by CHPs when distributing arPZQ. The approach was perceived as convenient, personalized, and effective in reaching all children in their homes.

*“First*, *he/she* [CHP] *is somebody whom I know so well*. *Secondly*, *it is more convenient since the CHP will bring the drug at the household level*, *as compared to walking to the hospital to pick the drug*.*” (Female FGD*, *Gonda)*

*“They [CHPs] should move around to cover the special groups in the community*.*” (Male FGD*, *Kandega B)*.

*“…Okay*, *if you are to compare interventions at the community level and intervention at the facility*, *you’ll find intervention done at the community yielding better results*. *So perhaps they [community members] would want someone to go to them because there would be a challenge of access to the facility… maybe transport challenges …*.*so I believe if it’s taken closer to them [community members]*, *then the uptake will be higher*.*” (KII*, *sub-county health records officer)*

Some participants preferred public health facilities. Fixed points such as markets and schools were least preferred.

*All participants: “Nobody will come*. *[to fixed points]*.*” (Female FGD*, *Remba)*

*“…*.*Go reach out*. *You do not wait for them [parents] to come*…*we used to have static places where we would sit at a market and wait for them to come*, *like for measles vaccines*, *they would not come*. *It forced us to go door-to-door and get them*.*” (KII*, *Malaria coordinator)*

#### Preferred social mobilization platforms

Similar to arPZQ delivery platforms, CHPs were the most preferred platform for social mobilization in all six islands, despite Sukru Island not having a CHP.

*“Here in our Island*, *the CHP knows how to pass information to all of us*.*” (Female FGD*, *Takawiri)*

*“CHP would be in a better position to do that*, *but we don’t have one*. *So if you could organize for us to get our own CHP*.*” (Female FGD*, *Sukru)*

Other preferred platforms for mobilization were the local radio station ‘*Ekialo kiona*,*’* beach management units, churches, and public address systems, with posters being the least preferred. All participants agreed that health messaging should focus on risks and treatment of schistosomiasis. There was also curiosity among parents about the new formulation’s (arPZQ) dosing, administration, and potential side effects, identifying the need to include these aspects of the drug in the social mobilization package.

*“The whole community should be taught more about the disease*, *how one can get it*, *how to prevent it*, *and how it can be treated*.*” (Female FGD*, *Ngodhe).*

*“The drug we have been taking in the past is normally given according to the height; I wish to know the criteria for this new formulation in terms of quantity and dosage*. *………*‥ *I wish to know the side effects of the drug before our children participate in the new formulation*.*” (Male FGD*, *Takawiri)*

*“Will you measure the child’s height and weight before giving out the drug*?*” (Female FGD*, *Wamai- Mfangano)*

### Community recommendations for a successful delivery of arPZQ in hard-to-reach settings

Community members and health personnel provided valuable recommendations to improve the delivery of arPZQ in hard-to-reach settings. Health promotion emerged as a top recommendation. Specifically, participants suggested raising awareness about risks and treatment of schistosomiasis and the impact of the disease on the community. Information about arPZQ, its significance, and potential side effects can empower individuals to make informed decisions about their children’s health.

“*Before we even focus on drugs, we have to give a background on how we get this disease*, *the effects of this disease on individual health*, *and the impact of this disease if not treated early*. *And then issues to do with signs and symptoms*, *and briefly*, *we come back to prevention*, *then about drugs and environmental sanitation combined with strategies for preventing schistosomiasis*.*” (KII*, *Community health services lead-Suba North)*

Timing for arPZQ distribution is vital. Community input highlighted the need to coordinate it with religious events, fishing seasons, and the school calendar to maximize participation among school-going children under 5 years. FGD participants suggested expanding arPZQ distribution days and involving more CHPs for efficient and extensive coverage of at-risk children. Most key informants advised integrating arPZQ distribution into existing health programs like immunization and mother-child programs to maximize resources, expand coverage, and ensure the sustainability and effectiveness of schistosomiasis control efforts.

*“We also face the challenge regarding the timing of the activity*. *For example*, *when the drug distribution is done on Sunday when people are in church*, *this may result in some community members missing the drug*.*” (Male FGD*, *Gonda)*

*“It [arPZQ distribution] would depend on the period*. *When it is school-going time*, *it is tricky*. *Okay*. *However*, *when the schools are closed*, *people are on holiday because nowadays they [children] go to school as young as one year*.*” (KII*, *Malaria Coordinator)*

*“I think the distribution period [days] and CHPs should be increased to ensure more people get the drug” (Male FGD*, *Gonda)*.

*“Through immunization programs*, *you will get them [PSAC] because we are giving them Malaria vaccine from 6 months*.*” (KII*, *Immunization officer)*

## Discussion

An interplay of social, economic, political, and cultural challenges [41] affect achieving the global targets for schistosomiasis control listed in the Neglected Tropical Disease (NTD) 2021–2030 Roadmap [42] and elimination efforts. In anticipation of arPZQ MDA pilots in selected countries and subsequent rollout, and with the objective of reaching all at-risk PSAC, there is a need to understand treatment-related contextual factors and identify the most suitable platforms to deliver arPZQ in hard-to-reach communities. In our study, we identified health system-level, population-level, and physical geographic barriers as the major hurdles to healthcare access in hard-to-reach settings.

This study offers a nuanced perspective on the level of knowledge about schistosomiasis among community members. The findings indicate that a substantial portion of the participants exhibited at least basic knowledge of schistosomiasis, including an understanding of its symptoms, preventive measures, and the side effects associated with treatment. It is conceivable to attribute this high awareness to the high prevalence of schistosomiasis in the study area [24, 28, 29]. The community has been targeted for MDA with praziquantel (PZQ) by the national control program in previous years, as part of the Kenya National Breaking Transmission Strategy (BTS) [43]. This strategic intervention has significantly contributed to raising community awareness about the disease in the study area. Our findings align with similar studies conducted in various regions, such as Kome Island in the north-western part of Lake Victoria [44], and Zanzibar islands [45] in Tanzania, and endemic communities along Lake Albert in Uganda [46]. Nonetheless, a study conducted on Lake Victoria islands in Uganda reported a notably lower level of knowledge about schistosomiasis among community members and healthcare staff [47]. It is imperative to acknowledge that our study revealed certain knowledge gaps, particularly the misinterpretation of symptoms and the disease’s specific transmission mechanisms. This observation resonates with findings from a study carried out under the SCORE project in Western Kenya [48]. Furthermore, the misconception of haematuria as a symptom of sexually transmitted infections only, as identified in our study, has also been documented in studies conducted in Magu district of Tanzania, situated at the shore of Lake Victoria [49], Western Kenya [48], Mozambique [50] and among caregivers in South Africa [51]. This can hinder control efforts [52] as affected persons may not seek medical care due to shame and stigma [49, 53]. Moreover, lower levels of knowledge on schistosomiasis often coincide with increased levels of misconceptions, which may lead to suboptimal prevention practices within a community [54–56]. Our findings underscore the importance of targeted health promotion and awareness campaigns to improve the understanding of both community members and CHPs, and enhance effective schistosomiasis prevention and control measures within hard-to-reach communities.

The association between exposure to freshwater bodies contaminated with human faeces and urine and schistosome infections has been well documented. Various studies have established that engaging in routine domestic activities, such as laundry and dishwashing, infants being bathed or seated in containers of freshwater while their caregivers perform household tasks, as well as activities like swimming and fishing in freshwater bodies within endemic regions, are closely associated with the transmission of schistosomiasis [12, 46, 57–60]. In our study area, the lake serves as the primary source of water and a significant source of livelihood, particularly through fishing activities. Consequently, community members frequently immerse themselves in waters potentially infested with schistosome cercariae. Additionally, open defecation practices are prevalent in this area, further exacerbating the contamination of freshwater bodies with schistosome eggs. These predisposing factors collectively contribute to the continued transmission and prevalence of schistosomiasis within the community. There is therefore a need for comprehensive interventions that address both the environmental and socio-economic determinants of schistosomiasis. Potential strategies encompass enhancing the water, sanitation, and hygiene infrastructure, alongside improving access to health promotion [61–63]. These multifaceted interventions aim to not only reduce the vulnerability of community members to schistosomiasis but also enhance their overall well-being.

In this study, participants identified several primary barriers to accessing healthcare services in hard-to-reach settings. The findings are aligned with prior research that has highlighted similar impediments to healthcare access. For instance, a study on access barriers to maternal healthcare services in selected hard-to-reach areas of Zambia [64], identified poor road networks as the main factor affecting access to health services. A systematic review focused on barriers to maternal care access in low-income African countries [65] identified challenges, including the quality and availability of healthcare services, physical and geographical hindrances, and socio-cultural and economic factors. Another review conducted in Namibia examined access barriers preventing children with disabilities from utilizing healthcare services, reported themes related to the scarcity of public health facilities and challenges arising from deficient transportation systems, road networks, and infrastructure, particularly in areas with limited resources [66]. These findings echoed similar challenges faced in rural regions of Malawi [67], Ghana [68], and Senegal [69].

The overarching context of schistosomiasis within the healthcare systems of endemic sub-Saharan countries is marked by neglect [41]. Consequently, national governments often do not prioritize the disease and allocate inadequate resources for its control and transmission interruption, particularly in marginalized hard-to-reach settings [70]. To achieve the objectives of universal health coverage and sustain the progress made thus far in the control of schistosomiasis, governments in endemic nations must engage in collaborative, multidisciplinary efforts [71]. This involves consolidating resources, promoting interdisciplinary cooperation, and integrating strategies to control and eliminate schistosomiasis. Notably, in Malawi, schistosomiasis control has been integrated into the Essential Health Package (EHP), which offers a comprehensive set of eleven basic health interventions to all Malawians [67, 72]. This integration demonstrates a commitment to addressing the neglected disease within a broader healthcare framework, with the potential to enhance community participation in schistosomiasis control efforts.

The barriers listed above collectively contribute to suboptimal healthcare seeking behaviors, particularly within hard-to-reach communities. In our study area, parents and caregivers typically sought formal medical treatment for their children only when the children’s health had deteriorated to a severe state or when such treatment was financially feasible. In many sub-Saharan African countries, this pattern of healthcare-seeking behavior is pervasive [45, 49, 73].To address these barriers and rectify existing health inequalities, particularly in the context of schistosomiasis control and elimination, new interventions are imperative.

Given the prevailing challenges stemming from infrastructural healthcare limitations and geographic constraints, especially in hard-to-reach areas, a door-to-door approach employing CHPs emerged as the preferred platform for delivering arPZQ and community education and mobilization. CHPs play pivotal roles as sources of treatment and health-related information across various illnesses, including schistosomiasis [74], in several African countries, including Kenya. In our study, CHPs were perceived as convenient providers of personalized care, effectively reaching all children in their homes. Evidence from Uganda supports the positive impact of involving CHPs in promoting health among preschool-aged children, leading to reduced malnutrition, decreased morbidity, and improved child health practices [75]. Similar impact of CHPs have been reported in the control programs for trypanosomiasis and onchocerciasis in Kenya and Nigeria [72].The knowledge of CHPs about the cultural norms, context, and language of their communities proves advantageous, allowing them to deliver health communication in a culturally sensitive and acceptable manner [72]. This, in turn, enhances the coverage of treatment and sensitization efforts. An evaluation of Tanzania’s onchocerciasis control program affirmed the pivotal role of community sensitization in the program’s success and sustainability [76]. However, based on the health service access challenges and limited knowledge on the disease’s specific transmission mechanisms among study participants in our study area, there is a pressing need to ensure an adequate number of well-trained CHPs equipped with knowledge related to schistosomiasis and its related aspects for a successful control program.

Throughout our study, community members displayed a predominantly positive outlook regarding the potential success of new health interventions, especially within the realm of schistosomiasis control. Their optimism was rooted in positive prior experiences with innovative healthcare initiatives, such as the malaria vaccine [77], favorable feedback from previous MDA campaigns with PZQ, resulting in their willingness to participate in future MDAs. For the successful acceptance of the arPZQ program in the challenging, hard-to-reach setting of our study area, participants emphasized the necessity of tailored health promotion. This education should focus on the risks of schistosomiasis, the benefits and potential side effects of arPZQ, targeted treatment, and strategic planning with consideration of timing, to ensure inclusivity. Previous research has emphasized the significance of incorporating a comprehensive health promotion component for the success of any MDA program [78–80].

### Limitation

Our study utilized a convenience sample, hence these findings can only be generalized to our study area and possibly to similar hard-to-reach settings in the African Great Lakes.

## Conclusion

This study highlights the paramount role of health system, population, and geographic barriers to healthcare access in hard-to-reach settings, perpetuating preexisting health disparities. Despite these challenges, we found a positive community outlook regarding the adoption and success of new health interventions, including arPZQ. In response to existing health systems infrastructural and geographic challenges in hard-to-reach settings, the door-to-door approach utilizing CHPs emerged as the favored delivery platform for arPZQ and health promotion. CHPs, with their intimate familiarity with local contexts and languages, also play a pivotal role in delivering culturally acceptable health communication, the need for which was underscored by our findings and must accommodate all at-risk populations in schistosomiasis prevention and control efforts.

## Supporting information

S1 Checklist Inclusivity in global research. (DOCX)

S1 File Case interview guide. (PDF)

S2 File Focus group discussion guide. (PDF)

S3 File Key informant interview guide. (PDF)
