# Supplementary material for: Contextual factors influencing schistosomiasis treatment and identification of delivery platforms for arpraziquantel in hard-to-reach areas and populations in Homa Bay County, Kenya
Source: PLOS Glob Public Health. 2024 Dec 19;4(12):e0004035. doi: 10.1371/journal.pgph.0004035 (PMC11658621; doi:10.1371/journal.pgph.0004035)
Supplement: S1 File — (PDF) [file pgph.0004035.s002.pdf]

Case Interview Guide
(For Parents/Guardians of Preschool-Aged Children)

County: ____
Sub-county: ____________________________________
Ward: ____________________________________
Date of the interview: ____
Name of the Moderator: _____________________________________
Name of the Notetaker/Recorder: _____________________________________
Venue of the discussion: ______________________
Start time: ________________ End time: _______________________

Instructions:

- This form should be used for case interviews with parents/guardians of preschool-aged children (PSAC).
- After obtaining written informed consent, read the following instructions to the participants.

Introduction

Good morning/afternoon. My name is................. I come from the Division of Vector Borne & Neglected Tropical Diseases, Ministry of Health. We are undertaking an assessment for community-based treatment activities in NTD programs. The main aim of this assessment is to assess the knowledge, attitude, and perceptions towards community-based health interventions across communities. Specifically, we plan to conduct a programme for children aged 2-5 years for the treatment of Bilharzia **with a new formulation of the standard drug - arpraziquantel 150mg – that is orally dispersible, smaller size, and with an improved taste,** in this County. I request you share your honest views on the issues we will discuss.

Your participation in this discussion is voluntary and you are free to stop your participation in this discussion if you feel uncomfortable at any point. I would like, however, to assure you that the information you provide shall be kept confidential and will only be used for the purposes of this study. This discussion will last approximately 45 minutes. I will moderate the discussion and record this conversation.

Do you have any questions or comments before we proceed?

Moderator: *(If any question/comment, please first address them before proceeding with the discussion).* We would like to request that we record your responses on this phone during the discussion because it would be difficult for the note-taker to record all the discussion points.
Obtain consent.

*Do not probe on '**possible themes'.***

Ice breaker

What are some of the challenges encountered in this community? **Probe on the following (if not mentioned during the discussion): Health, education, water, security, poverty, unemployment etc.**

Key Issues for Discussion

1. Knowledge, understanding and perceptions on schistosomiasis/bilharzia

i. How would you describe Bilharzia in this area?

Possible themes to note:
- ✓ *The local names*
- ✓ *Age and gender of most affected*
- ✓ *Risky behaviors*
- ✓ *Perceptions of people towards the diseases etc.*

ii. Are you aware of the signs and symptoms of Bilharzia?

Possible themes to note:
- ✓ *Diarrhea*
- ✓ *High fever*
- ✓ *Itchy skin/ rash*
- ✓ *Muscle/joint pain*
- ✓ *Abdominal pain*
- ✓ *Bloody stool*
- ✓ *Bloody urine etc.*

iii. Do you know of any preventive measures for Bilharzia?

Possible themes to note:
- ✓ *Swimming in the lake/chlorinated pool*
- ✓ *Drinking clean boiled/treated water*
- ✓ *Eating food washed with clean water*
- ✓ *Snail control by use of recommended chemicals*
- ✓ *Avoid contact with infected waters*
- ✓ *Proper human waste disposal – community-led total sanitation*
- ✓ *Deworming drugs etc.*

iv. What are the sources of treatment for Bilharzia for community members?

Possible themes to note:
- ✓ *Government health facilities*
- ✓ *Private health facilities*
- ✓ *Self-medication from the chemist*
- ✓ *Mass Drug Administration (MDA)*
- ✓ *Prayers, traditional healers/remedies etc.*

v. How would you describe your experience with previous treatment programmes in this community?

Possible themes to note:
- ✓ *Attitude of the healthcare providers*
- ✓ *Trust of the campaign treatments*
- ✓ *Perceptions on the drug (splitting, crushing/ mixing with water, taste)*
- ✓ *Knowledge – limited awareness creation and advocacy*
- ✓ *Past experience e.g. side effects*
- ✓ *Time and frequency of social mobilization and distribution etc.*

vi. As a parent living with a disability, what specific challenges do you encounter in accessing health services for your children?

Possible themes to note:
- ✓ *Physical accessibility of healthcare facilities*
- ✓ *Transportation difficulties*
- ✓ *Availability of disability-friendly services*
- ✓ *Communication barriers*
- ✓ *Attitudes of healthcare providers*
- ✓ *Need for assistive devices or support*

vii. What support do you think is necessary to better accommodate parents living with disabilities in health programmes?

Possible themes to note:
- ✓ *Accessible transportation options*
- ✓ *Home-based health services*
- ✓ *Training for healthcare providers on disability awareness*
- ✓ *Inclusive communication methods (e.g., sign language interpreters)*
- ✓ *Peer support groups*
- ✓ *Policy changes to support disability inclusion*

2. Now I would like us to talk about the treatment of Bilharzia among children aged 2-5 years in this area

i. We plan to conduct a treatment programme among children aged 2-5 years for treatment of Bilharzia in your area. What are the conditions under which the community, including parents living with disability, may be willing to participate in the campaign?

Possible themes to note:
- ✓ *Knowledge and awareness of the drug*
- ✓ *Drug administration challenges – access, duration, frequency, administration platforms*
- ✓ *Fears, myths, and misconceptions*
- ✓ *Negative past experience e.g. side effects*

ii. Where do you prefer to get information on Bilharzia treatment for children under 5 years old?

Possible themes to note:
- ✓ *Media–radio, posters, text messages, brochures, video clips, T.V, social media*
- ✓ *Healthcare Providers*
- ✓ *Community Health Volunteers (CHVs)*
- ✓ *Community Leaders*
- ✓ *Barazas/Community meetings*
- ✓ *Religious leaders*
- ✓ *Informal groups etc.*
- ✓ *Social media (Facebook, WhatsApp, Twitter etc.)*
- ✓ *Community coalitions made up of people who practice desired behaviors etc.*
- ✓ *Dance and concerts conveying key messages*

iii. What do you think should be emphasized so that the whole population is mobilized?

Possible themes to note:
- ✓ *Knowledge around the drug (e.g. safety, size)*

- ✓ *Drug administration procedures*
- ✓ *Reference to fears, myths, and misconceptions*
- ✓ *Consequences of refusal etc.*

iv. What methods of drug distribution do you prefer?

Possible themes to note:
- ✓ *Door-to-door by CHVs*
- ✓ *Fixed point i.e. health facilities schools/ECDs, religious institutions, marketplaces, etc.*
- ✓ *Child Health Day (Malezi bora)*
- ✓ *Combined approach using all the above distribution platforms etc.*

For each of the mentioned platforms, Probe the following:
- ✓ *Rumors/concerns/misconceptions*
- ✓ *Timing and frequency of distribution*
- ✓ *Who should distribute the drugs – CHVs, healthcare workers, teachers, etc.*

v. As a parent living with a disability, what challenges do you face in participating in community health campaigns?

Possible themes to note:
- ✓ *Accessibility of campaign venues*
- ✓ *Availability of information in accessible formats*
- ✓ *Support needed to attend (e.g., transportation, caregiver assistance)*
- ✓ *Perceptions and attitudes of the community towards parents with disabilities*
- ✓ *Communication barriers*

vi. How can community health campaigns be made more inclusive for parents living with disabilities?

Possible themes to note:
- ✓ *Providing materials in accessible formats (Braille, large print, sign language)*
- ✓ *Ensuring physical accessibility of venues*
- ✓ *Offering transportation or mobile services*
- ✓ *Training CHVs and healthcare workers on disability awareness*
- ✓ *Creating support networks for parents with disabilities*

3. Child health and family health decision-making

i. Who makes decisions about your children's health?

Possible themes to note:
- ✓ *Men (husbands, fathers)*
- ✓ *Women (wives, mothers)*
- ✓ *Extended family members (grandparents, uncles, aunties)*

ii. What do you do when your children aged 2-5 years fall sick?

Possible themes to note:
- ✓ *Waiting for a few days to see if the child feels better*
- ✓ *Home remedies*
- ✓ *Self-medication with available drugs at the house*
- ✓ *Visiting the public health facility*
- ✓ *Visiting the private health facility*
- ✓ *Ask a traditional healer*

- ✓ *Praying and blessings etc.*

4. Recommendations

What specific measures should be implemented to better support parents living with disabilities in accessing treatment for their children?

Possible themes to note:

- ✓ *Accessible healthcare facilities and services*
- ✓ *Disability-inclusive health policies*
- ✓ *Regular training for healthcare providers on disability inclusion*
- ✓ *Community sensitization to reduce stigma*
- ✓ *Provision of assistive devices and support services*
- ✓ *Creation of inclusive health communication strategies*

THANK THE PARTICIPANT
