# Supplementary material for: Contextual factors influencing schistosomiasis treatment and identification of delivery platforms for arpraziquantel in hard-to-reach areas and populations in Homa Bay County, Kenya
Source: PLOS Glob Public Health. 2024 Dec 19;4(12):e0004035. doi: 10.1371/journal.pgph.0004035 (PMC11658621; doi:10.1371/journal.pgph.0004035)
Supplement: S3 File — (PDF) [file pgph.0004035.s004.pdf]

Key Informant Interview (KII) Guide
(For Health authorities and Identified Gatekeepers in the community).

County: ____
Sub-county: ____
Ward: ____
Date of the interview: ____________________________________
Name of the interviewer: _________________________________
Venue of the discussion: __________________________________
Start time: ________________
End time: ____________________

Introduction
Good morning/afternoon. My name is................. I come from Homabay, Division of Vector Borne & Neglected Tropical Diseases, Ministry of Health. We are undertaking an assessment for community-based treatment activities in NTD programs. The main aim of this assessment is to assess the knowledge, attitude, and perceptions towards community-based health interventions across hard-to-reach communities of Mbita. Specifically, we plan to conduct a treatment programme for children aged 2-5 years for the treatment of Bilharzia **with a new formulation of praziquantel 150mg that is orally dispersible, smaller size, and has a better taste.** I request you to share your honest views on the issues we will be discussing.

As a health care worker/director/programme coordinator/ community gatekeeper, your involvement in this process has generated experiences as diverse as they are important, especially to improve the organization of activities related to this treatment.

Your participation in this discussion is voluntary and you are free to stop your participation in this discussion if you feel uncomfortable at any point. I would like, however, to assure you that the information you provide will be kept confidential and will only be used for the purposes of this study. This discussion will last approximately 45 minutes.

Do you have any questions or comments before we proceed?
Interviewer: *(If any question/comment, please first address them before proceeding with the discussion).*
Obtain consent/use of recorder (use of script/consent form)

*Do not probe on '**possible themes'.***

Icebreaker
What are some of the challenges encountered in this community?

Possible themes to note: Health, education, water, security, poverty, unemployment etc.

Key Issues for Discussion
1. About his/her position and role
Tell me about yourself and your role in this community:
- How long have you held your current role?
- What is your role in health interventions programs?
- Have you participated in Neglected Tropical Diseases' (NTD; e.g. schistosomiasis, soil-transmitted helminths) Program activities?

2. Now I would like us to focus this discussion on Neglected Tropical Diseases (NTDs) in this community.
i. How would you describe the Schistosomiasis/Bilharzia situation in this area?

Possible themes to note:
- ✓ *Prevalence and intensity/ burden of the disease*
- ✓ *Community knowledge and perceptions – uptake, misconceptions, fears etc.*
- ✓ *Treatment/Control, including among children aged 2-5 years*
- ✓ *Trainings done on schistosomiasis*
- ✓ *Safety of the drug etc.*

ii. How would you describe the problem of Schistosomiasis/ Bilharzia on children aged 2-5 years in Mbita?

Possible themes to note:
- ✓ *Risk of infection, burden of disease*
- ✓ *Drug administration - size and taste of the tablet (bitter taste, splitting, crushing, dissolving in water)*
- ✓ *Knowledge of caregivers*
- ✓ *Fear, rumors, and misconceptions*
- ✓ *Side effects etc.*

iii. What are preferred sources of treatment for Schistosomiasis /Bilharzia by community members?

Possible themes to note:
- ✓ *Government health facilities*
- ✓ *Private health facilities*
- ✓ *Traditional healers and home remedies*
- ✓ *Chemist/Pharmacy*
- ✓ *Mass Drug Administration (MDA) etc.*

3. Now I would like us to talk about treatment programmes in this county
i. What is your experience with different health programmes in this area?

Possible themes to note:
- ✓ *Uptake of various interventions - people not showing up, refusals, hesitancy*
- ✓ *Myths and misconceptions, lack information on treatment, the disease is not a priority*
- ✓ *Trust in the government programs*
- ✓ *Experiences on NTD programs*

ii. What are some of the challenges you have experienced during previous community treatment programmes, for example deworming, polio campaigns, malaria etc?

Possible themes to note:
- ✓ *Lack of Supplies*
- ✓ *Inadequate storage facilities*
- ✓ *Delay in delivering the drugs from the National pharmacy to the county pharmacy/ community*
- ✓ *Poor logistics and coordination*
- ✓ *Poor motivation/ lack of incentives of the health workers*
- ✓ *Increased workload to CHVs*
- ✓ *Terrain and weather conditions etc.*

iii. We plan to conduct a treatment programme for children aged 2-5 years for the treatment of Schistosomiasis/Bilharzia **(arpraziquantel 150mg – orally dispersible, smaller size, better taste)** in this area. In your view, how would you describe the community's (parents/guardians) willingness to take part in the campaign?

Possible themes to note:

- ✓ *Fears, myths, and misconceptions*
- ✓ *Past experience – side effects*

iv. How would you describe decision-making processes regarding child health in the community?

Possible themes to note:

- ✓ *Men (husbands, fathers)*
- ✓ *Women (wives, mothers)*
- ✓ *Extended family members (grandparents, uncles, aunties)*
- ✓ *Guardians/Caregivers*

v. In your opinion, which is the best channel for relaying information on Schistosomiasis /Bilharzia treatment for children aged 2-5 years to community members?

Possible themes to note:

- ✓ *Media–radio, posters, text messages, brochures, video clips, T.V, social media*
- ✓ *Healthcare Providers*
- ✓ *Community Health Volunteers (CHVs)*
- ✓ *Community Leaders/groups*
- ✓ *Barazas/Community meetings*
- ✓ *Religious leaders (Churches/Mosques)*
- ✓ *Informal groups*
- ✓ *Text messages*
- ✓ *Social media (Facebook, WhatsApp, twitter etc.)*

vii. To communicate these types of information, what do you think should be emphasized on so that the whole population is mobilized?

Possible themes to note:

- ✓ *Knowledge and awareness of the drug*
- ✓ *Perceptions on the drug*
- ✓ *Drug administration procedures*
- ✓ *Fears, myths, and misconceptions*
- ✓ *Safety of the drugs*
- ✓ *Refusal and hesitancy etc.*

vii**.** What is your preferred method of drug distribution in this area?

Possible themes to note:

- ✓ *Do door to door by CHVs*
- ✓ *Have a fixed-point i.e. health facilities, schools/ECDs, religious institutions, marketplaces, etc.*
- ✓ *Child Health Day (Malezi bora) etc.*
- ✓ *Combination all the above platforms – mixed approach etc.*

For each of the mentioned platform, probe on the following:

- ✓ *Rumors/concerns/misconceptions*
- ✓ *Timing and frequency of distribution*
- ✓ *Who should distribute the drugs – CHVs, healthcare workers, teachers etc?*

4. Recommendations

i. What key measures would you suggest should be put in place to ensure there is uptake of the new paediatric formulation - arPZQ 150mg by community members?

Possible themes to note:

- ✓ *Budget allocation for campaign treatment*
- ✓ *Political ownership and buy-in*
- ✓ *Incentives to the health workers involved*
- ✓ *Put policies and frameworks in place to support implementation*
- ✓ *Training of the health workers and CHVs who will be involved*
- ✓ *Proper and timely social mobilization*
- ✓ *Involvement of local structures*
- ✓ *Strengthen the supply chain management*
- ✓ *Robust monitoring and surveillance systems, research and knowledge sharing*
- ✓ *Trainings of programme implementers*
- ✓ *Supervision and coordination etc.*

THANK THE RESPONDENT
